# Decentralizing Clinical Tumor Exome Analysis: On-Premises GPU-Based Secondary Analysis in a Clinical Molecular Laboratory

**DOI:** 10.3390/genes17101186

**Published:** 2026-09-25

**Authors:** Pankaj Ahluwalia, Denton Lord, Mason Arbery, Navya Pampatwar, Aditi Mohan, Ashutosh Vashisht, Ashis K. Mondal, Ravindra Kolhe

**Affiliations:** Department of Pathology, Augusta University, Augusta, GA 30912, USA; pahluwalia@augusta.edu (P.A.); amondal@augusta.edu (A.K.M.)

**Keywords:** on-premises genomics, decentralized genomic analysis, data sovereignty, molecular diagnostics, solid tumor exomes, benchtop computing, tumor-only variant calling, colorectal cancer, GPU-accelerated variant calling

## Abstract

**Background/Objectives:** Clinical tumor sequencing has become central to precision oncology, yet secondary genomic analysis remains heavily dependent on cloud or centralized computational infrastructure, creating barriers related to cost, turnaround time, data governance, and accessibility. We demonstrate that secondary analysis of clinical tumor exomes can be performed entirely within a clinical molecular laboratory using workstation-class GPU hardware, eliminating dependence on external computational infrastructure. **Methods:** Sequence data generated off site were analyzed on premises with a variant-calling workflow run on benchtop GPU workstations. Accuracy was assessed against the Genome in a Bottle germline and Sequencing Quality Control Phase 2 (SEQC2) HCC1395 somatic reference materials and recall of the variants listed on the clinical molecular reports for 172 institutional colorectal tumor exomes. **Results:** We analyzed all 172 exomes from reads to called variants on device, at a median of 25.1 min of on-device compute across the 170 exomes with complete timing. On the germline reference material, an F-measure of 0.9963 was obtained genome-wide, and 0.9945 restricted to the exome target. Against those reports, we recovered all 153 reported variants across the four driver genes (KRAS, NRAS, PIK3CA, and BRAF). Coding-variant recall on this development cohort was 95.74% (1168/1220) requiring exact reference and alternate allele agreement, and 98.44% (1201/1220) when 33 indels matching on coding or protein position alone were also counted. Call sets were identical across triplicate runs of five exomes on both devices. **Conclusions:** Secondary analysis of solid tumor exomes was completed on workstation-class hardware in a colorectal cancer cohort, showing that clinical molecular laboratories can independently perform this step without reliance on external computing resources. The architecture, in which each site analyzes its own reads, can serve as a node in a federated genomic system.

## 1. Introduction

Genomic sequencing has become a routine component of solid tumor diagnosis and management [1]. Comprehensive tumor sequencing guides the selection of targeted therapy in patients with metastatic cancer [2]. In colorectal cancer specifically, RAS and BRAF mutation status informs eligibility for anti-EGFR therapy [3,4]. When sequencing is outsourced to an external laboratory, the downstream analysis is constrained by the computational infrastructure available to the center that receives the data. This molecular diagnostic work depends on secondary analysis, the step from sequence reads to called variants, in which cloud and off-site computing play a critical role. Although sequencing technologies have rapidly become more accessible, computational analysis remains centralized in cloud platforms or external computational environments. This dependence introduces additional costs, data-transfer requirements, vendor reliance, cybersecurity considerations, and delays that disproportionately affect smaller clinical laboratories and resource-limited healthcare systems.

Centralized analysis imposes substantial data-handling requirements. The rate at which sequencing data are generated has made large-scale computational infrastructure a prerequisite for their interpretation [5], and the cost of a genomic test extends beyond the sequencing reaction to the handling and analysis of the data it produces [6]. Adherence to guideline-recommended biomarker testing is incomplete and varies widely across practice settings [4,7,8,9]. In one multi-practice cohort, only 28% of patients who received anti-EGFR therapy had complete guideline-aligned testing [4]. In a real-world cohort of 2694 patients, timely comprehensive genomic profiling was associated with greater use of matched targeted therapy in advanced non-small-cell lung cancer [10], and delayed molecular results are a recognized barrier to timely care [11]. Bringing sequencing in house has been modelled explicitly as a means of reducing this delay [12]. Comprehensive genomic profiling is also less accessible in resource-constrained settings [13,14].

Genomic data sovereignty is becoming increasingly important as institutions seek to maintain control of patient genomic information while minimizing cybersecurity risks associated with cloud-based analysis. On-premises deployments have been implemented at individual academic and community centers, including rapid whole-genome and point-of-care sequencing services operating on local hardware [15,16,17]. Despite these deployments, GPU-accelerated variant calling remains characterized on datacenter and cloud platforms [18]. The vendor’s own benchmarks report substantial runtime reductions across cloud platforms and both short- and long-read sequencing technologies, with concordance maintained against established reference standards [19]. Further, comparable runtime reductions have been reported by independent benchmarks at equivalent datacenter scale [20]. Whole-genome germline single-nucleotide variant calling on a single workstation-class GPU has been reported at approximately one minute of turnaround, with 99.97% concordance against a CPU reference implementation [21].

On-premises secondary analysis of clinical tumor exomes has been constrained by available hardware capability and by the extent to which that capability can be scaled within a single institution. Whether modern workstation-class GPU systems can eliminate dependence on centralized computational infrastructure remains largely unexplored. Because each institution independently performs secondary analysis, this architecture is compatible with federated genomic networks, which retain data at each site and query across institutions [22,23]. We hypothesized that advances in GPU computing have reached the point where secondary analysis can be fully integrated within the clinical molecular laboratory, allowing institutions to retain complete control of genomic data.

Clinical-grade analysis carries a defined set of requirements. A workflow used for clinical reporting must establish analytical sensitivity and specificity for each variant class it reports, a limit of detection in allele fraction, a defined reportable range, and performance in regions where alignment and calling are difficult, together with software-change control, audit trails, quality assurance and regulatory accreditation. What follows reports feasibility, recall of variants that a clinical laboratory reported, and accuracy against public reference materials.

In this study, we utilized publicly available tools in a tumor-only variant-calling workflow and ran it on benchtop GB10 workstations (Figure 1). We assessed the workflow against the genome in a Bottle germline and SEQC2 HCC1395 somatic reference materials and against the clinical molecular reports for 172 institutional colorectal tumor exomes. The alignment, variant-calling and annotation steps employ tools and reference resources developed for solid tumors generally, independent of tissue of origin.

## 2. Materials and Methods

### 2.1. Specimens and Cohort

The workflow was evaluated on institutional colorectal tumor exomes profiled by a clinical reference laboratory; the raw sequence data were analyzed on premises. KRAS, NRAS, PIK3CA and BRAF are grouped throughout as driver genes because they carry the most reported variants in this cohort; RAS and BRAF status informs eligibility for anti-EGFR therapy in colorectal cancer [3,4], while PIK3CA is included on reporting frequency rather than on an equivalent guideline basis. The grouping is a reporting convention and not an actionability determination. Of 173 sequenced accessions, 1 was excluded because its sequence data failed quality control, giving 172 analyzed exomes, of which 171 carried at least one clinically reported coding variant. Those reports listed 1284 short variants in total, of which 1220 were coding single-nucleotide variants or indels; recall is scored on that coding set. The remaining 64 were splice-region, non-coding or silent changes. The reports contained no copy-number, fusion or expression calls. Matched normal specimens were not available, so all calling was tumor-only [24]. The reported set mixes somatic and incidental germline findings, and both are counted in the recall denominator. The cohort is 157 microsatellite stable, 14 microsatellite instability high and 1 indeterminate; tumor mutational burden is reported as low in 149, high in 21, indeterminate in 1 and absent in 1. Tumor purity, specimen type, sequencing batch and ancestry were not supplied with these specimens. The exome capture assay and its target interval are the property of the laboratory that performed the sequencing and cannot be released; coverage is, therefore, reported at the clinically reported loci rather than across the full capture target. The filter policy described below was developed on this colorectal cohort. The complete computational workflow was executed within the institutional network without cloud connectivity or external computational services.

At the clinically reported loci, per-sample median read depth was 2058x (10th–90th percentile, 977x–3515x) and the median variant allele fraction was 0.32 (10th–90th percentile, 0.14–0.62; minimum 0.03).

### 2.2. Analysis Platform and Workflow

The cohort was processed on two benchtop NVIDIA GB10 workstations, each with a 20-core Grace CPU and a Blackwell GPU sharing 121.6 GiB of unified memory. The two devices were used together to increase throughput.

Variant calling used NVIDIA Parabricks v4.7.0-1. Reads were aligned to the GRCh38 reference of the GATK resource bundle with BWA-MEM, then coordinate sorted and duplicate marked. Cohort calling was somatic and tumor-only, using Mutect2 (GATK 4.6.1.0) with the GATK best-practices somatic resources for GRCh38 (the 1000 Genomes panel of normals and the af-only gnomAD population allele-frequency resource). The germline caller used for the reference-material benchmark below was DeepVariant as implemented in Parabricks v4.7.0-1, run through the same alignment pipeline [25]. Alignment and variant calling ran on the GB10; annotation with the Ensembl Variant Effect Predictor (VEP) release-113.4 and all scoring ran on a separate on-premises x86 workstation, using the same containerized tool versions throughout.

### 2.3. Benchmarking and Concordance Assessment

Germline accuracy was measured against the Genome in a Bottle HG002 v4.2.1 benchmark on GRCh38 [26], using 35x PCR-free whole-genome reads from the NIST public repository, and scored with RTG Tools vcfeval v3.12.1 over the high-confidence regions. Reads for this benchmark were aligned to the GRCh38 no-ALT analysis set, the standard reference for scoring germline callers, not the ALT-containing reference used for the cohort. Somatic accuracy was measured against the SEQC2 HCC1395 reference [27]. Calls were run in tumor-only mode to match the cohort configuration, restricted to Ensembl protein-coding regions, and scored by position and allele with bcftools v1.9.

Concordance with the clinical reports was assessed at the filter setting described below and separately using the caller’s own filters alone. Calls were annotated and matched to a reported variant within the same specimen and gene, using a transcript-agnostic tiered match that took an exact Human Genome Variation Society (HGVS) coding change first, then an exact HGVS protein change. Exact allele agreement is the primary criterion, and recall on that basis is reported separately. For indels, a coding- or protein-position match without allele agreement was also recorded, and those matches are reported as a distinct tier rather than folded into the headline. Concordance is reported as recall of the clinically reported variants, with Wilson 95% confidence intervals overall, by variant class and by driver gene, computed per variant rather than per patient. A clinical report lists a curated subset of clinically relevant variants, not a complete call set; only recall against it is interpretable.

A call flagged only as germline is retained if its population allele frequency is at most 0.1%, as is an indel flagged only for short-tandem-repeat slippage or multiallelic sites; any other flag keeps it filtered. We used the conventional 0.1% rare-germline cutoff; recall was 98.93% at 1%, 98.44% at 0.1% and 96.89% at 0.01%. Ten-fold cross-validation chose 1% in every fold, and the conventional cutoff was kept at a cost of about half a percentage point of recall. Within each fold, the population allele-frequency threshold was the only quantity optimized, selected to maximize recall of the clinically reported coding variants on that fold’s training patients. Splitting was at the patient level, stratified by microsatellite status crossed with a tertile of reported variant count, so no patient contributed to both training and evaluation within a fold. The retention rules for short-tandem-repeat slippage and multiallelic indel flags were also set on this cohort. Because the policy was developed on this cohort, recall is also reported using the caller’s own filters alone and under cross-validation, and the headline is a development-cohort result.

Reproducibility across runs and across devices was assessed by processing five exomes, selected without formal criteria as a convenience sample, three times on each of the two GB10 workstations using the same software build. Agreement was assessed by comparing, for each run, an MD5 over the sorted chromosome, position, reference and alternate allele of every record passing filters. Genotype and quality fields were not compared. Every replicate of every exome produced the same value, and for each exome, the value was the same on both devices. Per-exome wall time for the GPU stages was recorded for 170 of the 172 exomes. The remaining two exomes lacked complete per-stage timing logs. Median per-stage times were 9.9 min for alignment and duplicate marking, 11.9 min for variant calling and 4.7 min for filtering. The two devices processed different specimens independently and did not cooperate on individual specimens; one read input from local disc and the other over a network mount, which carries roughly 6% read overhead and accounts for the difference between their medians. Annotation on the separate workstation was not timed per specimen, and queuing time, device utilization, peak memory, storage throughput, input read counts and total elapsed time for the cohort were not instrumented, so no end-to-end wall-clock figure is reported. No run failed or was retried.

## 3. Results

### 3.1. On-Device Runtime Across the Institutional Cohort

The institutional cohort comprised 172 colorectal tumor exomes, of which 171 carried at least one clinically reported coding variant, giving 1220 reported coding short variants scored for recall. Each exome was analyzed from sequence reads to called variants on a benchtop device, in a median of 25.1 min of on-device compute (interquartile range 21.4 to 29.6 min, range 5.6 to 45.5) across the 170 exomes with complete per-stage timing; the per-device medians were 26.1 min (*n* = 104, interquartile range 22.3 to 31.4) and 23.6 min (*n* = 66, interquartile range 19.8 to 28.0). The two devices processed different exomes independently rather than cooperating on individual cases; at the observed median, two devices running continuously correspond to approximately 115 exomes per day, which is a calculation rather than an observed production rate, since the cohort was not processed as a continuous run. No sequence data left the institution at any stage of the analysis, and all computation was performed within institutional infrastructure without dependence on external computational resources. Read alignment and variant calling ran on the benchtop device and annotation on a separate institution-owned workstation, all within the institutional network. Performance across the reference materials and the institutional cohort is summarized in Table 1.

### 3.2. Germline and Somatic Accuracy on the Reference Materials

On the Genome in a Bottle HG002 benchmark, which exercises the germline caller on whole-genome reads rather than the tumor-only path used clinically, the germline call set attained a precision of 0.9988, a recall of 0.9939 and an F-measure of 0.9963 across single-nucleotide variants and indels. Restricted to the exome target, the F-measure was 0.9945, with a precision of 0.9981 and a recall of 0.9910 over 22,800 benchmark variants.

On the SEQC2 HCC1395 somatic reference, run tumor-only and restricted to the exome target, we recovered 89.1% of single-nucleotide variants (423/475, 95% CI 85.9 to 91.6%) and 85.7% of indels (18/21, 95% CI 65.4 to 95.0%, on a small denominator). At this operating point, the single-nucleotide comparison comprised 423 true positives, 1370 false positives and 52 false negatives, giving a precision of 0.24, and the indel comparison 18 true positives, 119 false positives and 3 false negatives, giving a precision of 0.13. The tumor-only call set retains inherited variants of the specimen donor, which are scored as false positives against a somatic-only reference.

### 3.3. Concordance with the Clinical Molecular Reports

Across the 172 institutional exomes, which are the cohort on which the filter policy was developed, we recovered 95.74% of the clinically reported coding short variants requiring exact reference and alternate allele agreement (1168/1220, 95% CI 94.4 to 96.7%), and 98.44% (1201/1220, 95% CI 97.6 to 99.0%) when 33 indels matching on coding or protein position alone were also counted. Across all 153 reported variants in the four driver genes, KRAS, NRAS, PIK3CA, and BRAF, we recovered all 153, comprising KRAS 80/80, NRAS 8/8, PIK3CA 39/39, and BRAF 26/26 (Figure 2). All 17 reported BRAF V600E variants were recovered. Of the 153 driver-gene variants, 151 are coding and fall inside the 1220 scored above; the two splice-site variants do not.

In addition, the 33 matches that separate the two figures were made on coding or protein position without agreement of the reference and alternate alleles, and all of them are indels. They are listed individually in Supplementary Table S1.

Recall by variant class was missense 98.0% (598/610), nonsense 99.6% (246/247) and frameshift 98.3% (341/347); the remaining 16 reported coding variants fall in smaller classes and were all recovered (Figure 3). Of the 19 residual misses, 10 produced no call anywhere in the caller’s output, including filtered records; 6 were germline-flagged variants whose population frequency fell above the threshold used here; 2 were filtered as low-confidence; and 1 carried a haplotype flag.

Without 92. 46% (1128/1220, 95% CI 90.9 to 93.8%), 73 fewer variants than with filtering applied. Ten-fold cross-validation that reselected the population-frequency threshold on training patients chose the more permissive 1% cutoff in every fold, at which coding recall is 98.93% (1207/1220, 95% CI 98.2 to 99.4%). On the independent somatic reference material, filtering raised single-nucleotide recall from 420/475 to 423/475.

At the 0.1% threshold, the median per-exome candidate-variant list a laboratory would review alongside the clinical report was 4180 (interquartile range 3484 to 5315).

### 3.4. Reproducibility Across Replicate Runs and Devices

Five exomes were reprocessed three times on each of the two devices. All three replicates agreed for every exome on both devices, with no discordant calls, and the two devices produced the same call sets.

## 4. Discussion

Genomic sequencing is an established component of solid tumor care [1]. The associated secondary analysis is computationally demanding, and the scale of sequencing data has made large-scale computing infrastructure a prerequisite for its interpretation [5]. Workstation-class GPU hardware has begun to reach this scale, with whole-genome germline single-nucleotide variant calling reported in approximately one minute on a single device [21]. Federated architectures have separately been used to distribute analytic capability across multiple institutions [22,23]. We, therefore, characterized the extent to which workstation-class hardware supports this analysis. On benchtop GB10 devices, the workflow processed all 172 institutional colorectal tumor exomes, at a median of 25.1 min of on-device compute across the 170 with complete timing logs, and no sequence data were transmitted outside the institution. On the Genome in a Bottle reference material, the germline call set attained an F-measure of 0.9963; that benchmark exercises the germline caller on whole-genome reads and speaks to the hardware and the alignment path rather than to the tumor-only workflow used clinically, for which the SEQC2 HCC1395 experiment is the closer proxy and the weaker result. We recovered all 153 reported variants in the four driver genes, and 95.74% of reported coding variants on exact allele agreement, 98.44% including indels matched on position alone. Three of those genes, KRAS, NRAS, and BRAF, inform eligibility for anti-EGFR therapy in colorectal cancer [3,4]. These results indicate that a clinical molecular-diagnostics laboratory can perform secondary analysis on site using a workstation-class device.

Four variants first scored as missed had in fact been called and had passed filters. Each was written differently on the clinical report and by the caller: a nine-base insertion recorded once as an insertion and once as the equivalent duplication, a pair of adjacent substitutions called as a single two-base variant, and a two-base variant the caller emitted as two phased substitutions. One of the four is coding and falls inside the 1220 scored for recall; the other three are splice-site variants outside it. The candidate-variant list generated by the workflow is substantially larger than the curated variant set enumerated on the clinical report. At the threshold used here, the median per-exome list was 4180 on-target candidates, against a mean of 7.5 reported variants per case (1284/172).

Both sequencing and secondary analysis can now be performed within a single institution. Local sequencing services are already in operation at academic and community centers [16,17]. Improvements in compute have brought the analysis step onto a device of the class used here. The cohort was processed across two benchtop devices operated in parallel, indicating that throughput can be increased by adding devices. Replicate runs of the same reads returned the same call sets on both devices. Each institution performs its own secondary analysis and retains control of the patient’s genomic data (Figure 1).

The clinical report lists a curated subset of variants, so concordance with it measures recall rather than accuracy. The filter policy was developed on this cohort and awaits testing on an independent one. It rests on population allele frequency, and population databases cover non-European genomes less completely, so performance varies with ancestry [28]. Ancestry, tumor purity, specimen type and sequencing batch were not supplied with these specimens. These variables act on tumor-only calling and on the frequency filter, and a larger study can measure them. Tumor-only calling without a matched normal retains the inherited variants of the individual, which are counted as false positives against a somatic-only reference and set a precision ceiling that local compute does not change.

Historically, secondary genomic analysis represented one of the major barriers preventing smaller molecular laboratories from implementing comprehensive genomic profiling. Advances in workstation-class GPU hardware now allow computationally intensive analyses to be performed entirely within individual clinical laboratories rather than requiring centralized cloud infrastructure. Maintaining genomic data entirely within institutional infrastructure may simplify compliance with institutional privacy policies, cybersecurity requirements, and evolving national regulations governing genomic data sharing. Further, continued gains in GPU capability and in the accompanying software would be expected to shorten the compute this workflow requires. Establishing secondary analysis within the molecular diagnostics laboratory would strengthen its capability and bring an essential step of patient care closer to the patient. Performing secondary analysis locally removes the dependence on external computational infrastructure for that step, and institutions retain their analytical workflows, computational resources and software versions. No economic analysis was performed in this study. As workstation-class computing continues to evolve, this approach could be extended beyond tumor exomes to whole-genome sequencing, transcriptomics, methylation profiling, liquid biopsy analysis, structural variant detection, and multimodal AI-assisted molecular diagnostics.

This study is a research demonstration rather than a validated clinical assay. A workflow used for clinical reporting has to establish more than concordance with variants that were already reported. It requires orthogonal confirmation of reported calls, a validated reportable range, an established limit of detection in allele fraction, characterized performance in regions where alignment and calling are difficult, documented software-change control, audit trails covering every analytical run, a quality-assurance program with defined acceptance criteria, and accreditation under the applicable regulatory framework. The results establish that the computation can be performed on a benchtop device inside the institution and that the calls it produces recover most of the variants a clinical laboratory reported on the same specimens.

## 5. Conclusions

Secondary analysis of clinical tumor exomes was completed on workstation-class hardware, at a median of 25.1 min of on-device compute per exome across the 170 with complete timing logs, with recall of 95.74% of the clinically reported coding variants across the 172-exome development cohort on exact allele agreement, 98.44% including position-only indel matches. Independent implementation and verification would be required for the workflow to be applied at other laboratories. Secondary analysis currently sent off site can be performed by a laboratory equipped with hardware of this class, which places the schedule for that step under the laboratory’s own control. Where profiling is timely, it has been associated with greater use of matched targeted therapy [10]. The alignment, variant-calling and annotation steps are independent of tissue of origin, so the same analysis applies wherever next-generation sequencing is used in diagnosis. Advances in workstation-class GPU computing have shifted the computational requirements for secondary genomic analysis. Our findings demonstrate that clinical molecular laboratories can independently perform secondary analysis of tumor exomes entirely on premises, with the recall of clinically reported variants and the accuracy against public reference materials set out above. This decentralized architecture bears on data sovereignty and laboratory independence, and federation remains a possible future direction.

## Figures and Tables

**Figure 1:**
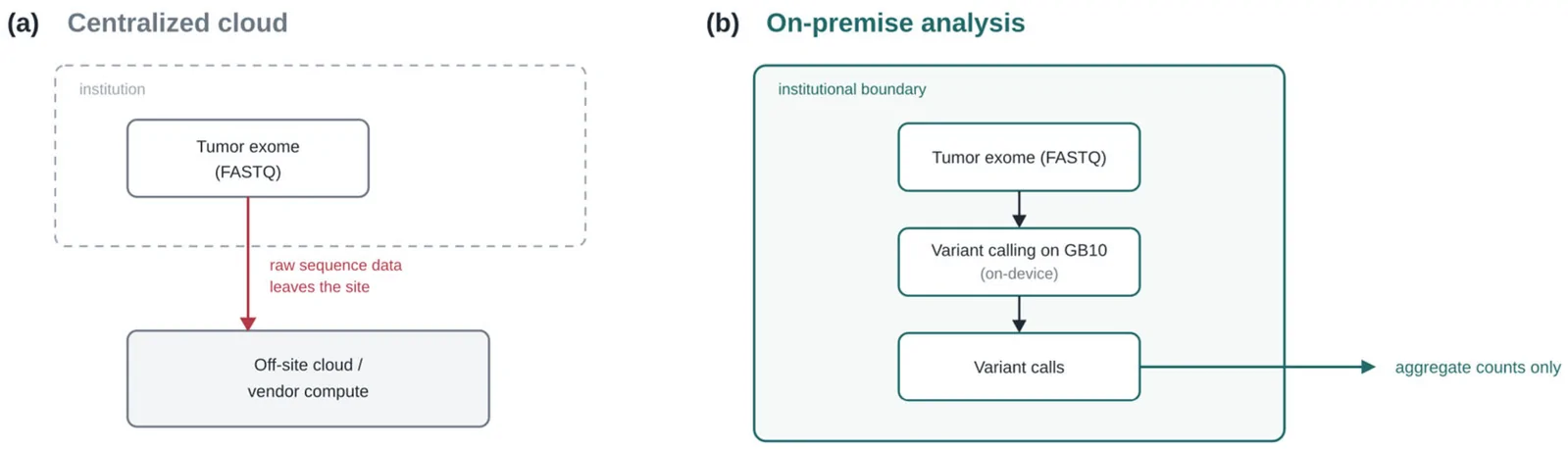
Architecture of centralized versus on-premises secondary analysis. (**a**) Reads are sent off site to cloud or vendor infrastructure. (**b**) The deployment tested here processes tumor exome reads from FASTQ to called variants on the benchtop GB10 within the institutional boundary. Because each site holds its own reads and shares aggregate counts alone, the architecture can serve as a node in a federated genomic network.

**Figure 2:**
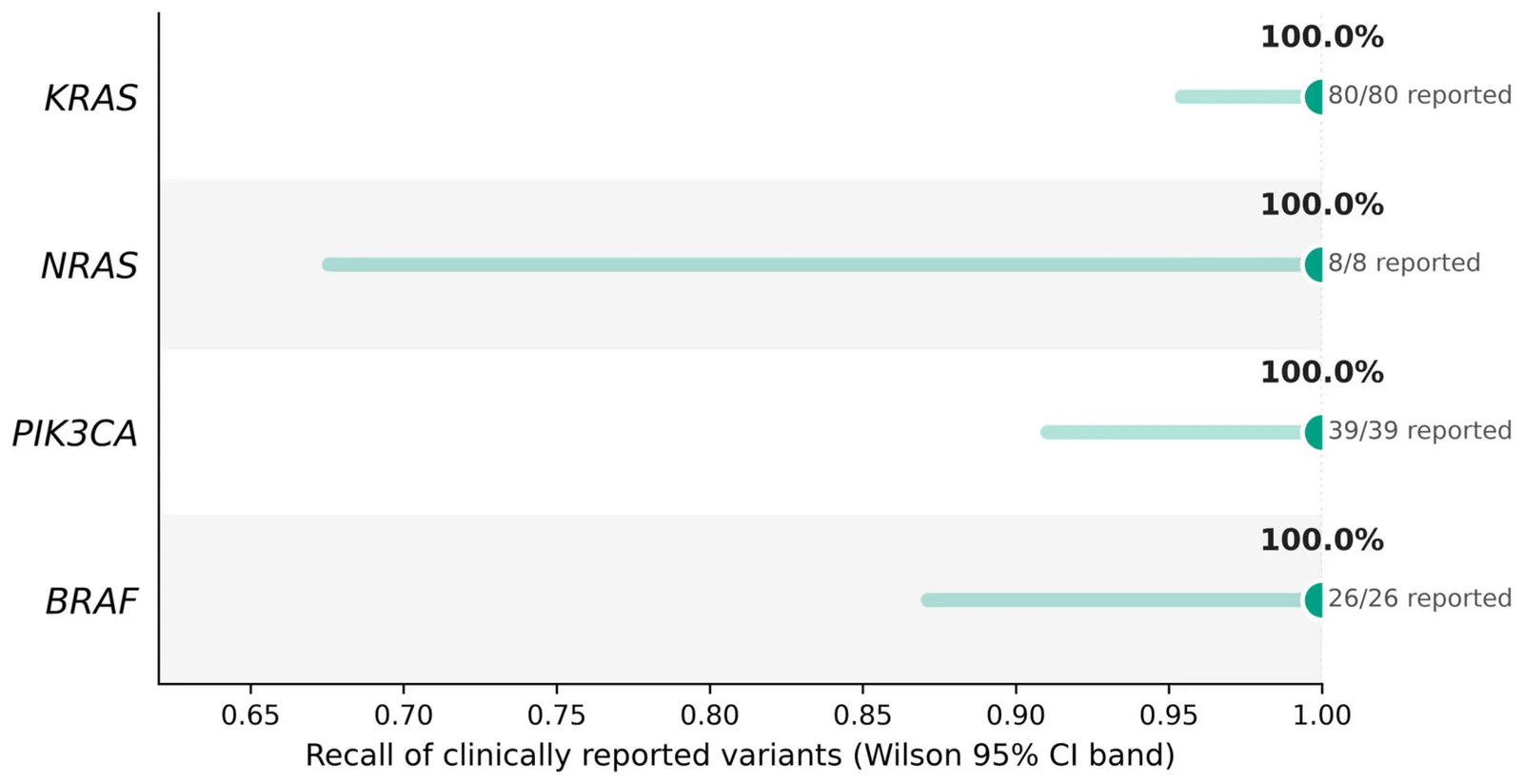
Recall of clinically reported variants in the four colorectal driver genes across the 172 tumor exomes. Per-gene point estimates with Wilson 95% confidence intervals: KRAS 80/80, NRAS 8/8, PIK3CA 39/39, BRAF 26/26.

**Figure 3:**
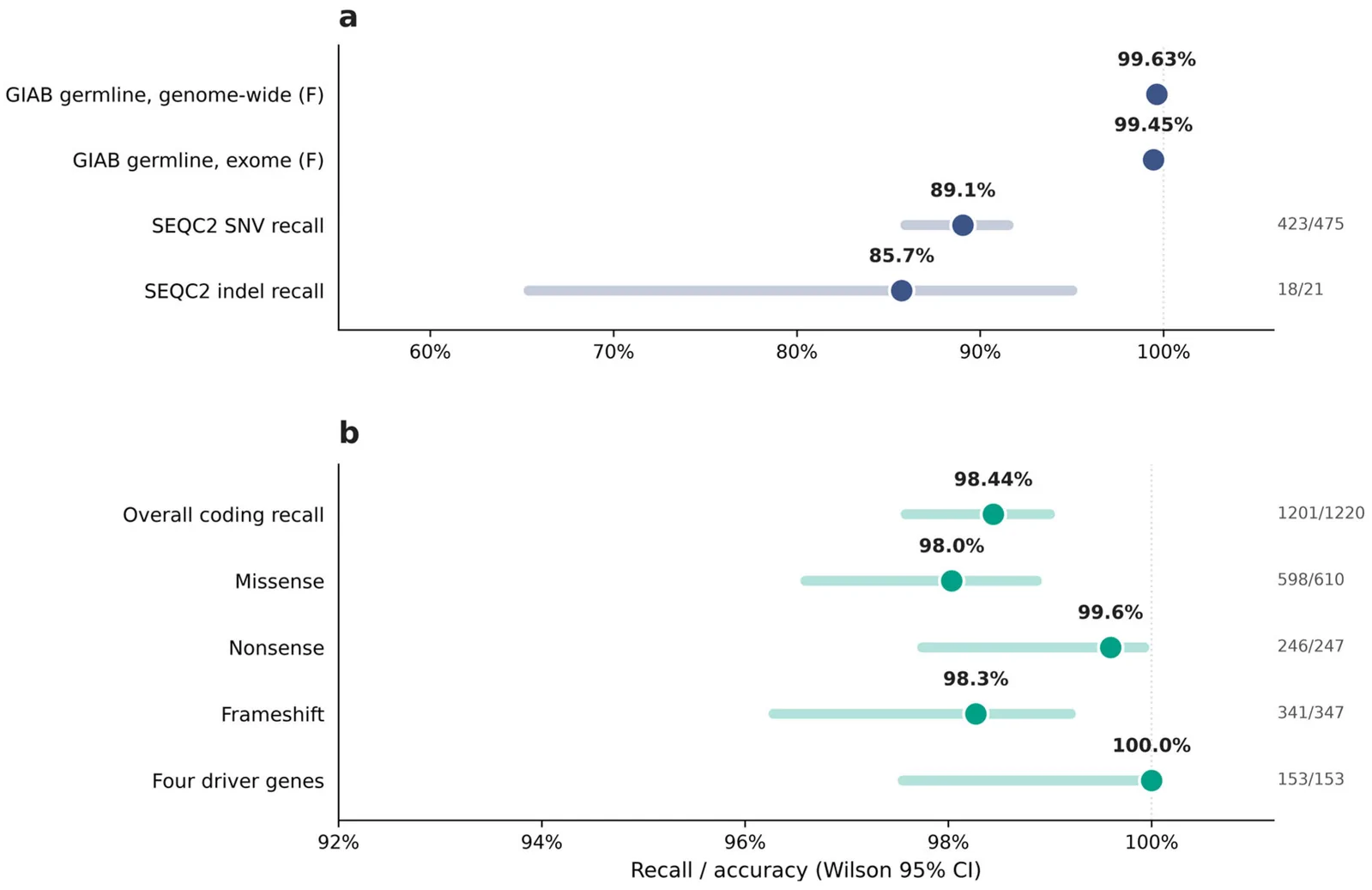
Accuracy and concordance summary. (**a**) Accuracy on the reference materials: Genome in a Bottle HG002 germline F-measure 0.9963 genome-wide and 0.9945 on the exome target; tumor-only SEQC2 HCC1395 somatic recall 89.1% (423/475) for single-nucleotide variants and 85.7% (18/21) for indels. (**b**) Concordance with the clinical molecular report across the 172-exome cohort: coding recall 95.74% (1168/1220) on exact allele agreement and 98.44% (1201/1220) including position-only indel matches, and 100% (153/153) across the four driver genes. Points carry Wilson 95% confidence intervals, except the two composite F-measures.

**Table 1:** Pipeline performance summary. Concordance is recall of the coding short variants listed on the cohort’s clinical molecular reports. Accuracy is measured against the Genome in a Bottle germline and SEQC2 HCC1395 somatic reference materials.

Metric	Value	Diagnostic Interpretation
Cohort	172 exomes	171 carried at least one reported coding variant
Coding recall vs. clinical report	95.74% (1168/1220) exact allele; 98.44% (1201/1220) including position-only	coding single-nucleotide variants and indels; the two rows differ by 33 indels matched on position alone
Recall in the four driver genes	100% (153/153)	KRAS 80/80, NRAS 8/8, PIK3CA 39/39, BRAF 26/26; all 17 reported BRAF V600E variants recovered
Recall by variant class	missense 98.0%, nonsense 99.6%, frameshift 98.3%	missense 598/610, nonsense 246/247, frameshift 341/347
GIAB HG002 germline	F-measure 0.9963	germline caller accuracy on a public reference material, measured on this device
SEQC2 HCC1395 somatic, tumor-only	SNV recall 89.1% (423/475), indel 85.7% (18/21)	somatic recall in tumor-only mode, on cell-line reference material
SEQC2 tumor-only precision	SNV 0.24, indel 0.13	precision in tumor-only mode against a somatic-only reference

## Data Availability

The public benchmark datasets are available from their original repositories, the Genome in a Bottle HG002 reference and the SEQC2 HCC1395 tumor/normal reference. The institutional colorectal tumor exome data are available from the corresponding author on reasonable request, subject to institutional data-sharing agreements and patient privacy. The workflow is deposited in the Zenodo research-data repository at https://doi.org/10.5281/zenodo.22755808, with its command lines, resource versions and container images. Open-source components are version-pinned in Section 2. The accelerated variant-calling toolkit is free for academic use under the vendor’s terms and is not open-source software.

## Supplementary Materials

The following supporting information can be downloaded at: https://www.mdpi.com/article/10.3390/genes17101186/s1. Table S1. Reported variants matched on position alone. The 33 indels counted as concordant on coding or protein position without exact allele agreement, across the 172-exome cohort.
